# Time to Re-evaluate the Guideline Value for Manganese in Drinking Water?

**DOI:** 10.1289/ehp.10316

**Published:** 2007-07-25

**Authors:** Karin Ljung, Marie Vahter

**Affiliations:** Institute of Environmental Medicine, Karolinska Institutet, Stockholm, Sweden

**Keywords:** children, drinking water, infants, infant formula, neurotoxicity

## Abstract

**Objective:**

We reviewed the scientific background for the current health-based World Health Organization (WHO) guideline value for manganese in drinking water.

**Data sources and extraction:**

The initial starting point was the background document for the development of the WHO’s guideline value for manganese in drinking water as well as other regulations and recommendations on manganese intake levels. Data referred to in these documents were traced back to the original research papers. In addition, we searched for scientific reports on manganese exposure and health effects.

**Data synthesis:**

The current health-based guideline value for manganese in drinking water is based partly on debatable assumptions, where information from previous reports has been used without revisiting original scientific articles. Presently, preparation of common infant formulas with water containing manganese concentrations equivalent to the WHO guideline value will result in exceeding the maximum manganese concentration for infant formula. However, there are uncertainties about how this maximum value was derived. Concurrently, there is increasing evidence of negative neurologic effects in children from excessive manganese exposure.

**Conclusions:**

The increasing number of studies reporting associations between neurologic symptoms and manganese exposure in infants and children, in combination with the questionable scientific background data used in setting the manganese guideline value for drinking water, certainly warrant a re-evaluation of the guideline value. Further research is needed to understand the causal relationship between manganese exposure and children’s health, and to enable an improved risk assessment.

Manganese is an essential element for all living organisms and occurs naturally in soil, water, and plants. The neurotoxicity of manganese after high occupational exposure by inhalation has been well documented. Manganism, a Parkinson-like disorder was first noted in 1837 in five pyrolusite mill workers and has since been reported in hundreds of occupationally exposed workers (Cook et al. 1974; Greenhouse 1971; Langauer-Lewowicka and Kujawska 1974; Levy and Nassetta 2003; Smyth et al. 1973). Manganese is often regarded as one of the least toxic metals by the oral route because homeostasis limits the gastrointestinal absorption. However, there is increasing evidence of neurotoxicity by the oral route especially in infants. They have a more sensitive nervous system than adults, and their homeostasis is not fully developed. While breast milk generally contains low manganese concentrations, significant amounts can be found in infant formula. Moreover, because infant formula is normally sold in powdered form, the manganese concentration of the water with which the formula is mixed may contribute significantly to the infant’s daily manganese exposure.

Manganese occurs naturally in both surface and groundwaters, as a result of weathered and solubilized manganese from soil and bedrock. Manganese is also deposited into waters from human activities. According to the Geological Survey of Sweden (SGU, unpublished data), manganese concentrations in Swedish ground-water used for drinking water are on average 150 ± 510 μg/L (median 60 μg/L), with maximum values as high as 30,000 μg/L. Around 20% of the 12,000 sampled wells had manganese concentrations exceeding the Swedish recommended guideline value of 300 μg/L. The U.S. Environmental Protection Agency (U.S. EPA 2003) reported median manganese concentrations in groundwater at 5 μg/L, with the 99th percentile at 2,900 μg/L. In urban areas, the median groundwater concentration of manganese was found at 150 μg/L, with the 99th percentile at 5,600 μg/L. In public water systems supplied by groundwater, approximately 3% of the 982 sampled sources exceeded the U.S. health reference level of 300 μg/L (U.S. EPA 2003).

## The Current Manganese Guideline Value for Drinking-Water Quality

The World Health Organization (WHO) has recently lowered the guideline value for manganese in drinking water from 500 to 400 μg/L. The previous guideline value of 500 μg/L was originally set in 1958 and was at the time based on the distinct impairment of water potability by excessive manganese concentrations (WHO 2004). This value was retained in the 1963 and 1971 international standards as a maximum allowable or permissible manganese concentration. In the first edition of the WHO guidelines for drinking-water quality published in 1983, the guideline value was lowered to 100 μg/L based on the staining properties of manganese. In the second edition from 1993, the guideline value was again raised to 500 μg/L, this time based on health motives (WHO 2004). The third and current edition of the WHO guidelines for drinking-water quality was published in 2006 and presented a health-based guideline value of 400 μg Mn/L (WHO 2006).

The current health-based guideline value of 400 μg/L for manganese in water is based on an estimated no observed adverse effect level (NOAEL) for manganese in food. To allow for the possible higher bioavailability of manganese when ingested with water than with food (WHO 2004), the NOAEL of 11 mg/day was divided by an uncertainty factor of three. Using an adult body weight (bw) of 60 kg, a tolerable daily intake (TDI) of 60 μg Mn/kg bw was derived. On the assumption that 20% of the TDI would be allowed to come from drinking water, and that an adult consumes 2 L water/day, the value of 400 μg/L was set as a health-based guideline value for drinking water (WHO 2004).

### How the NOAEL for manganese was established

In the background document for the WHO drinking-water guidelines (WHO 2004), the use of 11 mg as a NOAEL is based on a review by Greger (1999) and on a report on dietary reference values for manganese published by the Institute of Medicine in 2000 (IOM 2001):

A review of typical Western and vegetarian diets found average adult manganese intakes ranging from 0.7 to 10.9 mg/day (Greger 1999; Institute of Medicine [IOM] 2002 [*sic*]). The upper range manganese intake value of 11 mg/day from dietary studies is considered a NOAEL. It is not believed that this amount of manganese in the diet represents an overexposure to the element (IOM 2002 [*sic*]). (WHO 2004)

The IOM in turn, also refers to Greger (1999):

A NOAEL of 11 mg/day of manganese from food was identified based on the data presented by Greger (1999). Greger (1999) reviewed information indicating that people eating Western-type and vegetarian diets may have intakes as high as 10.9 mg/day of manganese [. . . .] Because no adverse effects due to manganese intake have been noted, at least in people consuming Western diets, 11 mg/day is a reasonable NOAEL from food. (IOM 2001)

However, the article by Greger (1999) did not focus on manganese intake from different diets but rather on potential biomarkers of manganese in human nutrition and toxicology. In the introduction section on manganese exposure she stated that “the average intakes of adults eating Western-type and vegetarian diets in various surveys ranged from 0.7–10.9 mg Mn/day,” with reference to Gibson (1994) and Freeland-Graves (1994). However, no mention of these values is made in Gibson (1994), whose article is a review on trace elements in vegetarian and omnivorous diets, with a concern for deficiencies in vegetarian diets. The Freeland-Graves (1994) article is also a review published in a book on risk assessment of essential elements by the International Life Sciences Institute. The intake values of 0.7–10.8 mg Mn/day were observed for Canadian women in a study carried out by Gibson and Scythes in 1982. These values were also cited in the Freeland-Graves (1994) review and presented in a table of daily manganese intakes compiled from several studies.

The values used for setting the NOAEL thus originate from one study, where 100 Canadian women aged 30 ± 6.1 years were asked to complete dietary protocols of all consumed foods and beverages (including drinking water) in their own homes for 3 consecutive weekdays (Gibson and Scythes 1982). The authors present both calculated and analyzed intake values. The calculated daily manganese intake ranged from 0.7 to 10.8 mg, where 90% of the women ingested < 5 mg/day, and almost half the women (40%) ingested < 2.5 mg/day. The average daily manganese intake was calculated at 3.1 ± 1.5 mg. The analyzed manganese intake from duplicate portions provided a slightly lower average daily manganese intake of 2.4 mg/day. The analyzed maximum intake was not presented. The slight discrepancy between the calculated and the analyzed manganese intakes was explained by the authors to be a result of an overestimation in portion size (Gibson and Scythes 1982).

The NOAEL of 11 mg/day thus is based on calculated daily intakes of manganese and not on actual measurements of manganese intakes. No mention is made of the subjects’ health statuses or why it seems unfounded to draw any conclusions on a “no observed adverse effect level” of daily manganese intakes at 11 mg. In fact, several studies from different countries have reported daily manganese intakes after 1982, when the Gibson and Scythes study was published. Table 1 shows manganese intake data from an array of countries and ages. Children’s average manganese intake from omnivorous diets was reported to be about 2 mg/day (mean values range from 1.3 to 3.6 mg/day), whereas adult omnivorous diets resulted in a slightly higher intake of about 2.7 mg/day (mean range 1.5–3.9 mg/day). This value is similar to the analyzed intake value of 2.4 mg/day reported by Gibson and Scythes (1982). Both children and adults with a vegetarian diet have reportedly higher values of daily manganese intake than those with an omnivorous diet, which can be explained by the higher manganese concentration in plants than in meat and fish. The daily intake also seems to differ with country of residence, likely because of differing diets. None of the studies found intakes as high as 11 mg/day.

## Manganese Metabolism in Children

Although the NOAEL for manganese used in the development of the drinking-water guideline value does not seem to have significant scientific basis, the current guideline value for drinking water likely is low enough to protect the health of adolescents and adults. The reason is that ingested manganese is subject to delicate homeostatic control, as it is an essential element. Only a small fraction (1–5%) of ingested manganese is normally absorbed from the gastrointestinal tract. The uptake is regulated so that when dietary manganese levels are high, the gastrointestinal absorption is reduced. Manganese is also rapidly cleared from the blood in the liver via excretion in bile (Aschner et al. 2005; Klaassen 1996).

Infant absorption and excretion, however, seems to differ from that of adults, although exact data on human infant manganese absorption is scarce because of the difficulties in achieving accurate determinations. Dörner et al. (1989) performed measurements of infant absorption by using a conventional balance technique and found that around 20% of the manganese in formula-fed infants was absorbed. This value is considerably higher than those found for adults. The difference may be due to the lower gastrointestinal pH and prolonged emptying rate of newborns compared with those of adults as well as the immature gastrointestinal tract of newborns where immature epidermis and increased skin hydration may facilitate a higher absorption (Agency for Toxic Substances and Disease Registry 2000; Milsap and Jusko 1994).

Concurrently, manganese retention is higher in infants than in adults. According to Lönnerdal (1994) the bile flow is low in infants, which may result in a lower excretion of manganese via bile and higher tissue retention. Moreover, certain tissue sites have a high affinity for manganese, and although these sites are saturated in adults, they strongly retain manganese in infants. Both hair and blood show decreasing manganese concentrations with age. Hatano et al. (1983) reported 3- to 4-fold higher blood manganese concentrations in 1-month-old infants than in adults. These results are supported by Rükgauer et al. (1997), who found a linear decrease in serum manganese concentrations with age when investigating 137 individuals ranging in age from 1 month to 18 years. Alarcon et al. (1996) also found a decrease in blood manganese concentrations with age when examining 180 infants ranging in age from 5 days to 12 months, although the decrease was not as marked as for the Japanese infants in the Hatano study. In support, Sakai et al. (2000) reported decreasing hair manganese concentrations with increasing age of 418 children and adolescents ranging in age from 6 months to 20 years.

The decrease in manganese concentration with age is likely due to both higher absorption and lower excretion of manganese in early childhood. Infants are also likely to have a higher manganese concentration at birth. Studies on manganese concentrations in cord blood have reported significantly higher concentrations than that found in the maternal blood. Rossipal et al. (2000) and Krachler et al. (1999) found that the manganese concentration in umbilical cord serum was 150% higher than that found in the corresponding maternal serum. Takser et al. (2003, 2004) reported geometric mean manganese concentrations in cord blood collected from 222 newborns in 2003 and from 87 newborns in 2004 at 39μg/L (range, 15–93 μg/L) and 34 μg/L (range, 17–90 μg/L), respectively. These values can be compared with a reference range of 5.0–12.8 μg/L in blood of 100 healthy adult volunteers in France assessed by Goulle et al. (2005), who also found a median concentration of 7.6 μg/L of manganese in blood.

Consequently, infant ingestion of manganese has to be regarded with a different approach than adult ingestion. Both their physiology and their behavior influence the extent of exposure and any potential negative health effects. It is thus very difficult to accurately extrapolate studies on adults to children. Similarly, it is unlikely that the NOAEL for manganese used in the development of the current drinking-water guideline is relevant to child and infant exposure.

### Neurotoxicity of manganese

In addition to a higher absorption and lower excretion of manganese in infants compared with that in adults, infants also have a more sensitive nervous system. Although manganese is an essential element that is needed by the infant to support normal brain growth and development (Dorman et al. 2006), a series of recent studies report links between excessive manganese exposure and neurologic disorders in children, mainly in the form of behavioral effects. Ericson et al. (2006) reported an association between the behavior of twenty-seven 11- and 13-year olds and prenatal manganese exposure, whereas Takser et al. (2003) found negative relationships between cord blood manganese levels and attention, nonverbal memory, and hand skills at 3 years of age. No significant relationships were found, however, at 6 years of age. A number of studies have reported relationships between manganese exposure from drinking water in particular and child behavior: A 10-year-old boy who consumed drinking water with a manganese concentration of 1,200 μg/L for a period of 5 years showed below average performance on memory tests but normal results on IQ and cognitive tests. The boy had elevated whole blood, urine, and hair manganese concentrations (Woolf et al. 2002). Manganese-related effects on neurobehavioral performance were also reported by He et al. (1994) and Zhang et al. (1995), who examined 92 matched-pair 11- to 13-year olds. The manganese concentration in the drinking water consumed by the exposed group of children was 240–350 μg/L compared to 30–40 μg/L for the control group. The exposed children had elevated hair (He et alet al. 1994) and blood (Zhang et al. 1995) manganese concentrations. They had lower scores on neurobehavioral tests (He et al. 1994) and also lower scores in mathematics and language (Zhang et al. 1995). Another study of the relationship between decreased intellectual functioning and manganese exposure through drinking water is reported by Wasserman et al. (2006). They found a dose–response association between well-water manganese concentrations and test scores of performance as well as verbal ability in one hundred forty-two 10-year-old children. The average manganese concentration in the wells was 800 μg/L and ranged from 4 to 4,000 μg/L. It should be noted that the water was sampled 2 years before the neurologic evaluation of the children.

Differences in behavior (hyperactivity, oppositional behavior) were also reported in children with different manganese concentrations in their tap water (Bouchard 2007). The study included 46 children, of whom 28 lived in houses with elevated manganese levels (average 600 μg/L) in the tap water. The water in the houses of the remaining children had average manganese concentrations of 160 μg/L. The children in the first group had elevated hair manganese concentrations, which correlated with the water manganese concentration, suggesting that manganese in tap water was a significant exposure source.

Relationships have thus been found for different biomarkers, different cognitive tests, and different sources of exposure. Although no single study is entirely convincing as to the level of concern for toxicity in infants and young children, the number of indicative studies makes it reasonable to assume that children are at considerably higher risk for toxicity than older individuals. It is presently unclear whether manganese exposure affects both younger and older children or if negative symptoms in older children are an effect of infant exposure. In most studies involving older children, the children could well have been exposed since early life, which does not exclude induction of a neurotoxic effect already during brain development.

The indicative results on neurobehavioral effects from excessive manganese exposure in humans are supported by an experimental study on infant rhesus monkeys fed infant formula with varying manganese concentrations. Golub et al. (2005) observed that infants receiving soy formula (300 μg Mn/L) and soy formula with added manganese (1,000 μg Mn/L) were engaged in less play and more passive affiliation than the control group receiving cow’s milk (50 μg Mn/L). The formula groups also had shorter wake cycles and shorter periods of daytime inactivity. Rodent studies have found that manganese is accumulated in the infant brain to a higher extent than in the adult brain (Dorman et al. 2000; Erikson et al. 2004; Takeda et al. 1999). However, mixed findings have been reported regarding manganese brain distribution and responses to manganese exposure in rodents (Brenneman et al. 1999; Pappas et al. 1997; Tran et al. 2002). According to Dobson et al. (2004), the behavioral changes observed in both infant humans and rhesus monkeys from excessive manganese exposure are not replicable in rodents.

## Infant Exposure to Manganese

So far, we have acknowledged that infants seem to be more susceptible to neurotoxic effects of excessive manganese exposure, that their manganese absorption and excretion differ from that of adults, and that the scientific basis of the current guideline value for drinking-water quality is questionable. But to what degree are infants exposed to manganese through drinking water?

Children who are breast-fed have a low exposure to manganese, as breast milk is their main source of nutrient intake and little manganese is excreted through breast milk. According to published literature, average manganese concentrations in human milk range from 3.1 to 7.5 μg/L, with higher concentrations found in the colostrum (Al-Awadi and Srikumar 2000; Krachler et al. 2000; Leotsinidis et al. 2005). Parr et al. (1991) reported manganese concentrations in human milk collected from mothers in Guatemala, Hungary, Nigeria, Philippines, Sweden, and Zaire. The median manganese concentrations were similar in Sweden, Hungary, and Guatemala at 3–4 μg/L. In Zaire and Nigeria, the median manganese concentrations were slightly higher—11 and 16 μg/L, respectively, whereas human milk from the Philippines had the highest median concentration at 40 μg/L. These differences were attributed to geochemical differences rather than to socioeconomic factors.

Many children, however, are fed with infant formulas usually purchased in powdered form and that must be mixed with water before feeding. Hozyasz and Ruszczynska (2004) analyzed manganese concentrations in a number of formulas intended for infants younger than 2 months. They found that standard formulas contained on average 330 ± 301 μg/L (range, 66–856 μg/L); hypoallergenic formulas based on partially hydrolyzed cow’s milk proteins contained 92 ± 52 μg/L (range, 29–173 μg/L); and special thickened infant formula held 618 ± 531 μg/L (range 89–1,152 μg/L). According to infant formula producers Nestlé, Semper, Mead Johnson Nutritionals, Nutricia, and Novartis, the manganese concentration of their infant formulas intended for children younger than 4 months is between 25 and 600 μg/L (Ljung et al. 2007). The higher concentrations are found in some formulas intended for infants who are allergic or intolerant to breast milk or who suffer from reflux (hydrolyzed milk whey/protein and soy products), whereas the lowest concentrations are found in some formulas intended for all infants (cow’s milk based). However, most formulas, both standard and allergenic, have manganese concentrations of about 400 μg/L. Compared with breast milk, most infant formulas thus contain approximately 100-fold higher manganese concentrations, not including any additional manganese from the water with which it is mixed. The quality of the water used for preparing infant formula is therefore essential for the well-being of infants. This was pointed out in the rolling revision of the WHO drinking-water guideline value where Sievers (2005) specifically articulated safety concerns regarding the reconstitution of infant formula with water containing high manganese concentrations. She also stated that when powdered formulas containing the average manganese concentration of 325 μg/L are mixed with drinking water containing manganese equivalent to the WHO guideline value, the maximum value for manganese in infant formula set by the European Commission’s Scientific Committee on Food (SCF 2003) is exceeded.

### Upper limit and adequate daily intake of manganese for infants

The tolerable upper limit (UL) of a nutrient is the “highest level of daily intake that is likely to pose no risk of adverse health effects in almost all individuals” (IOM 2001). Neither the SCF, nor the Food and Nutrition Board IOM were able to set a tolerable upper limit for manganese intake in infants younger than 1 year, reportedly because of the considerable degree of uncertainty on negative health effects. The authors of the reports also express a concern regarding the inability of infants to handle excess amounts of manganese and state that the intake source should be from food and formula only to prevent high intake levels (IOM 2001; SCF 2003). IOM has set a UL for adults above 19 years of age at 11 mg/day by dividing the estimated NOAEL of 11 mg/day with an uncertainty factor of 1, based on the lack of evidence of human toxicity from lower doses. For younger age groups (from 1 year to 18 years), the UL for adults was extrapolated and adjusted on the basis of relative body weight using reference weights (IOM 2001).

The adequate daily intake (AI) of a nutrient is the level at which a sufficient nutrient intake is provided. The IOM set an AI of manganese at 3 μg/day for infants younger than 7 months. The value was calculated by multiplying the average infant milk consumption (0.78 L/day) with the average manganese concentration in human milk (3.5 μg/L), because it is assumed that human milk provides adequate manganese amounts to exclusively breast fed infants (IOM 2001). The AI value thereby reflects the observed mean manganese intake of infants exclusively fed human milk from well-nourished mothers.

For infants between 7 and 12 months of age, the IOM set the AI at 600 μg/day, that is, markedly higher than that for infants younger than 7 months. It was based on older children’s additional manganese intake from complementary foods (IOM 2001). The IOM report refers to a study by Gibson and DeWolfe (1980) in which the average manganese intakes in 6- and 12-month-old infants were 71 and 80 μg/kg, respectively. By using reference weights of 7 and 9 kg, the total average manganese intakes were calculated at 500 and 720 μg/day, respectively, and by extrapolating the NOAEL for adults (11 mg/day), an average daily manganese intake of 567 μg was attained. By using these two approaches, an adequate daily intake of 600 μg/day was set (IOM 2000). We have not been able to find the original publication by Gibson and DeWolfe (1980) that addresses why it is difficult to have an opinion on the usefulness of their results in determining an adequate daily intake value. However, in the U.S. Food and Drug Administration’s total diet study by Egan et al. (2002), the manganese intake in infants 6–11 months of age was calculated at 410–580 μg/day. Although the difference between the AI set by IOM and the upper intake range calculated by Egan and co-workers may be negligible, the calculated intake of a 6-month-old infant is significantly less than the calculated adequate daily intake of 600 μg/L set by IOM.

### Maximum value for manganese in infant formula

In 2003, SCF published a revision of essential requirements of infant formula (to be used in the first 4–6 months of life) and follow-on formula (to be used by infants older than four months) (SCF 2003). The recommended nutrient intake values were based on a reference infant of 5 kg with a daily consumption of 0.78 L formula containing 500 kcal (100 kcal/kg bw/day). A maximum manganese concentration of 100 μg/100 kcal was recommended for both infant and follow-on formula. The maximum value was motivated by the following statement:

In spite of the absence of a well identified UL for manganese in infants, there is increasing evidence of the neurotoxicity of high exposure to manganese. Therefore, a maximum manganese content of 100 μg/100 kcal is proposed, which is below the estimated LOAEL in adults for manganese in water (4.2 mg/L). (SCF 2003)

It is not clear how the maximum value was derived, but the same line of reasoning was used in the setting of the maximum manganese value in infant formulas by the Life Sciences Research Office (LSRO), a U.S.-based nonprofit research organization. The LSRO also recognized the increasing evidence of manganese toxicity from high exposures and stated that “the proposed maximum of 100 μg/100 kcal is significantly below the estimated LOAEL in adults for manganese in water” (Raiten et al. 1998).

LOAEL is the lowest observed adverse effect level, and there are some questions regarding the accuracy of its application in setting the maximum value and the interpretation of the original results from where it is derived. According to Velazquez and Du (1994) the LOAEL was based on the findings of neurologic symptoms similar to those of Parkinson disease in people older than 50 years, who consumed water with a manganese concentration of 1,800–2,300 μg/L for older than 10 years (Kondakis et al. 1989). The LOAEL for the Kondakis study was identified as 2 mg/L, which corresponds to 0.06 mg/kg/day (Velazquez and Du 1994). On the assumption that a 70-kg person consumes 2-L per day, the U.S. EPA set the drinking-water LOAEL at 4.2 mg/day (0.06 mg/kg multiplied by 70 kg) (Greger and Malecki 1997). Note that the U.S. EPA refers to milligrams per day and the SCF refers to milligrams per liter. This miscalculation results in the use of an LOAEL more than twice the manganese concentration that was found in the drinking water resulting in neurologic symptoms in people older than 50 years. If instead the LOAEL based on body weight had been used (0.06 mg/kg/day), it would have resulted in 300 μg/day for a 5-kg infant, 420 μg/day for a 7-kg infant, and 540 μg/day for a 9-kg infant. On the assumption that a 5-kg infant consumes 0.78 L/day, the LOAEL for manganese in formula would then be 385 μg/L, which corresponds to 60 μg/100 kcal. It should also be noted that the Kondakis study reported findings on manganese intake in adults older than 50 years, and not on infants. Table 2 presents the maximum values for infant formula and the adequate daily intake values for manganese together with calculated LOAEL and intake values for different age groups and in different units.

In addition to referring to the estimated LOAEL, the LSRO motivated the maximum value of 100 μg/100 kcal, corresponding to 650 μg Mn/L, by stating that it is far beyond the range likely to be encountered in both milk- and soy based formulas. However, this is not entirely true because, according to the producers, infant formulas contain between 25 and 600 μg/L, depending on the brand, and analyses have found between 30 and 1,150 μg Mn/L (Hozyasz and Ruszczynska 2004; Krachler et al. 2000, 1998). These concentrations were found without additional manganese from drinking water.

Thus, it can be concluded that the maximum content of manganese in infant formula is not based on scientific data on infants but is instead based on studies of adults, which also seem to have been misinterpreted. It should also be noted that the maximum daily manganese intake from formula (500 μg/day) is about 2 orders of magnitude higher than the calculated adequate daily intake of 3 μg manganese for infants up to 6 months of age, which is based on the concentration in breast milk. The bioavailability of manganese in drinking water and formula is lower than that in breast milk, which may justify a higher concentration. However, it is questionable whether a 100-fold higher manganese concentration in formula is justifiable.

## Rounding Up

So, what do we know about the scientific background of the current drinking-water guideline value so far? We know this:

- The present WHO guideline value for manganese in drinking water is 400 μg/L.
- The guideline value is calculated for adults older than 19 years, presumably weighing 70-kg and drinking 2 L water per day.
- The guideline value is set based on an NOAEL of 11 mg Mn/day.
- The NOAEL originates from a study in which the authors discuss an overestimation of the calculated daily manganese intake and where only 10% ingested > 5 mg Mn/day.
- No comments are made on possible toxic effects in the referenced study or why it is incorrect to refer to a daily intake of 11 mg as an NOAEL.
- The average daily manganese intake is similar regardless of nationality, and although a vegetarian diet may result in higher daily intakes, the intake rarely exceeds 5 mg/day.

## Time to Re-evaluate the Current Guideline Values for Drinking Water?

The number of questionable assumptions in the development of the manganese guidelines, both for drinking water and for infant formula, in combination with the increasing number of reports on infant neurotoxicity from manganese exposure, certainly warrants a re-evaluation of the current guideline values, although the need for further research is obvious. This is an urgent task, as elevated concentrations of manganese in drinking water are quite common in most countries and an increasing number of people depend on groundwater for drinking purposes. A re-evaluation of the background data used in the calculations of the guideline value would by itself lower the value. Although a lower value may not necessarily be required for the majority of the population, sensitive groups such as infants and possibly also elderly may be at risk with the present guideline value for drinking water. Especially children receiving formula, which generally have greatly elevated manganese concentrations in comparison with breast milk, may be at risk for neurotoxic effects if the water used for preparing their food contains additional manganese.

The importance of good drinking-water quality for the well-being of children and infants has been pointed out in the rolling revision of the WHO drinking-water guideline value (Sievers 2005). Regarding manganese, Sievers (2005) specifically articulated safety concerns regarding the reconstitution of infant formula with water containing high manganese concentrations. Presently, the maximum value for manganese in infant formula is exceeded when mixing common powdered formulas with water containing manganese equivalent to the WHO guideline value. In addition, the maximum value itself is not based on reliable scientific grounds.

## Figures and Tables

**Table 1:** Daily manganese intake values in children and adults of different nationalities.

Reference	Age (years)	*n*, sex	Diet	Country	Mn intake (mg/day ± SD)
Gibson et al. 1985	1.8–2	36 M + F	Omnivore	Canada	1.5 ± 0.7
Pennington et al. 1986	2		Omnivore	United States	1.5
Schlage and Wortberg 1972	2.8–13	NS	Omnivore	Germany	1.8 ± 0.4
Gibson 1994	3–6	75 M + F	Omnivore	Ghana	2.0 ± 0.5
Aung et al. 2006	3–6	NS	Omnivore	Japan	1.3 (0.9–1.7)^b^
Smit Vanderkooy and Gibson 1987	4–6	106 M + F	Omnivore	Canada	2.4 ± 0.9
Leblanc et al. 2005	3–14	NS	Omnivore	France	1.9 (3.5^a^)
Cavan et al. 1993	5–8	136 M + F	Omnivore	Guatemala	3.6 ± 2.0
Gibson 1994 Gibson et al. 1989	4–7	21 M + F	Lactoovoveg	Canada	4.3 ± 1.4
Gibson 1994	4–6	62 M + F	Veg + fish	Malawi	2.7 ± 0.5
Gibson et al. 1991	6–10	67 M + F	Veg + fish	Papua New Guinea	3.7 ± 1.9
Roychowdhury et al. 2003	~10		Veg	India	4.9
Ellen et al. 1990		NS	Omnivore	Holland	3.3
Bro et al. 1990	30–34	100 M	Omnivore	Denmark	3.9
Leblanc et al. 2005	> 15	NS	Omnivore	France	2.3 (4.3)^a^
Aung et al. 2006	28–40	24 F + 1M	Omnivore	Japan	1.5 (1–4.1)^b^
Bocio et al. 2005		Estimated	Omnivore	Spain	2.4
Biego et al. 1998		Estimated	Omnivore	France	2.5
Roychowdhury et al. 2003		Estimated	Veg	India	4.9

Abbreviations: F, female; Lactoovoveg, lactoovovegetarian; M, male; NS, not specified; Veg, vegetarian.

a 95th percentile.

b Range.

**Table 2:** Guideline and calculated values for manganese presented for different age groups and in different units.

			Manganese concentration		
Parameter	Age (months)	Assumed weight (kg)	μg/100 kcal	μg/L	μg/day
Max value infant and follow-on formula	0–12		100^a^	650	500
Adequate intake	0–6	5		3.8	3^b^
	7–12	7–9		600	600^b^
LOAEL	0–6	5		385	300
	7–12	7–9		420–540	420–540
	Adult	70		2,000	4,200^c^
Intake from formula	0–6	5		650	500
	7–12	7–9		700–900	700–900

a Data from SCF (2003),

b IOM (2001), and

c Velazquez and Du (1994).
